# Evaluating Pharmacy Student Perspectives and Attitudes Towards Compliance Aids and Devices Through Health Disparity Simulation

**DOI:** 10.3390/pharmacy13020054

**Published:** 2025-04-10

**Authors:** Bradley Phillips, Jason Powell

**Affiliations:** Department of Pharmacy Education and Practice, College of Pharmacy, University of Florida, 1225 Center Dr., Gainesville, FL 32610, USA; jpowell@cop.ufl.edu

**Keywords:** simulation-based training, type 2 diabetes, ambulatory care, health disparity, compliance aid/device

## Abstract

Objective: This study intends to evaluate simulated experiences provided to pharmacy students that directly compare the perspective of patients managing chronic disease states through traditional means without compliance aids to those using compliance aids, such as continuous glucose monitors (CGMs) and other devices. Methods: This simulation was conducted with third-year pharmacy students enrolled in the ambulatory care elective course at the University of Florida College of Pharmacy. It was designed to simulate a patient responsible for self-administering an array of medications for multiple chronic diseases that the students are likely to encounter during clinical practice. For the first week, students were tasked with adhering to a complex medication schedule from their associated pill bottles without the use of compliance aids (pill organizers, alarms, etc.) and checking their blood glucose twice daily using a traditional glucometer. In the second week, students continued the role of the patient; however, they were provided with compliance aids and encouraged to set alarms and use CGMs. Using a questionnaire developed based on the traditional Likert scale model, the students were able to quantify their experiences in a way that allowed the investigators to observe any changes. Results: Regarding the overall implications of this experience, most participants (>80%) agreed that this project increased their understanding of the value of compliance aids and devices and encouraged them to not only incorporate them into their future patient care plans but also advocate for accessibility to improve health outcomes. Conclusion: Students who completed this experience reported better adherence to chronic disease state control using compliance aids and, in turn, the applicability of the use of compliance aids in managing those with complex medication regimens. This simulation may encourage future pharmacists to incorporate compliance aids with their patients to improve health outcomes.

## 1. Introduction

The management of chronic disease states has historically been a challenging task for patients, caregivers, and healthcare providers. More than 35 million Americans are diagnosed with Type 2 Diabetes (T2DM), and the management of T2DM can be particularly challenging as it relies on the diagnosed individual to self-monitor their blood glucose, manage complex medication regimens, and make lifestyle changes that are sometimes difficult to incorporate in their typical daily life [1,2]. To add an additional barrier, underserved patient populations are not able to obtain the same quality of care resulting from several factors, including lower access to healthcare professionals along with the inability to obtain appropriate therapies, devices, and compliance aids (a device or tool utilized to optimize medication administration). This was highlighted by the American Diabetes Association publishing a statement acknowledging the benefits of using a continuous glucose monitor (CGM) and how these devices continue to be difficult to obtain for poorer patients, Black and Brown Americans, as well as those on government insurance [3]. When providing instruction to pharmacy students, it is often difficult to convey how impactful these disparities can be in the management of chronic disease states.

Studies have been published attempting to educate students via simulation-based training and the utilization of compliance aids for chronic disease state management [4]. Pill organizers are still one of the most commonly used compliance aids despite a possible lack of evidence of their benefit [5]. Most studies incorporate the use of a pill organizer to enlighten students on the barriers to adherence and provide a perspective of patient adherence in relation to patient outcomes [6,7,8]. However, these studies only provide one perspective with the incorporation of a compliance aid and do not directly compare the difficulties between the management of chronic disease states with and without such devices in the same cohort. For the T2DM component, there have been studies that display similar outcomes to the medication adherence studies, creating experiences for students to empathize with T2DM patients and understand the difficulties and perceptions of this patient population [9,10,11,12,13,14,15,16]. One study published in 2016 was conducted with the primary objective to determine the impact of a T2DM immersion experience on students’ perception of adherence difficulty for medication utilization and self-monitoring [16]. The authors were able to quantify the difficulty in managing T2DM; however, this was conducted prior to the rise in CGM availability and, again, does not highlight comparisons to those with and without said devices.

There is currently a lack of multimodal simulated experiences that directly compare those attempting to manage chronic disease states through traditional means and those using compliance aids and devices within the same student cohort. This leaves an outlet to develop an appropriate learning environment combining disease-state management immersive experiences and the realization of healthcare disparities and lack of adequate resources. This educational experience aimed to enhance pharmacy students’ viewpoints and attitudes by simulating the management of chronic diseases with and without compliance aids and devices.

## 2. Materials and Methods

This simulation was conducted with thirty-five third-year pharmacy students enrolled in the ambulatory care elective course at the University of Florida College of Pharmacy. At the beginning of the elective, students were provided with a complex ambulatory care patient that they would simulate throughout the duration of the course. The simulation was designed to imitate a patient having an array of medications, and multiple chronic disease states that the students would encounter during rotations and clinical practice. For the first week, students were tasked with adhering to a complex medication schedule from their associated pill bottles without the use of compliance aids (pill organizers, alarms, etc.) and checking their blood glucose twice daily using a traditional glucometer. Each student was provided with ten appropriately labeled prescription bottles filled with different types of candy associated with the medication they were prescribed to take. Medication treatment regimens were aimed at prompt administration considerations including, but not limited to, multiple daily dosing, alterations in daily dosing throughout the week, different routes of administration (i.e., injectable therapy), and administration timing considerations to avoid drug/dietary interactions. Students were informed of what candy types were included to avoid allergies or food insensitivities. They were also provided with all necessary supplies to perform self-monitoring of their blood glucose, including diabetic testing supplies, training on using said testing supplies, and a packet of patient-friendly educational materials similar to what a patient would receive from their healthcare provider. Students were instructed to check their blood glucose twice daily within designated time windows (10–11 a.m.; 6–7 p.m.) and acknowledge their compliance within the course site. Students were not responsible for reporting the actual blood glucose value received but simply needed to select that they checked their blood glucose. To minimize the bias of grade dependence facilitating blood glucose acknowledgement, a threshold was set where students would only need to check their blood glucose ≥ 60% of the time throughout the duration of the simulation to receive full credit. Students were not penalized for submitting a late blood glucose check outside of the designated time windows; however, they were encouraged to consider the factors that led to their delay in checking their blood glucose.

For the second week, students continued the role of the patient; however, they were provided with a compliance aid and device (pill organizer and CGM). Students were instructed to fill out and submit a picture of their pill organizer prior to conducting their medication administration for the week. Students were also encouraged to use other forms of medication compliance aids, such as setting alarms or timing administration with daily routine activities. For the CGMs, students had the same daily glucose checking requirement previously stated within the designated time windows. The students were also trained and educated in person in applying and interpreting the CGM by a medical science liaison from Abbot Diabetes Care Incorporated^®^. To appropriately simulate the functionalities of a CGM to a non-diabetic pharmacy student, blood glucose alerts were provided through the course site to the students in real time, and they were required to respond with appropriate action. For example, students received a simulated alert through the course site stating that their blood glucose is currently 67 mg/dL and were provided with answer choices on how to respond appropriately. After the conclusion of the second week, students were responsible for submitting a reflection on their experience. They were also asked to submit a voluntary survey to exemplify their thoughts and attitudes within three domains: glucose monitoring, medication adherence, and overall implications of a patient managing their chronic disease states with and without the use of compliance aids and devices pending descriptive statistics tests to quantitatively interpret the results. Using a questionnaire developed based on the traditional Likert scale model, the students were able to quantify their experiences in a way that allowed the investigators to observe any changes. This study was granted IRB exemption prior to conducting the research.

## 3. Results

**Figure 1:**
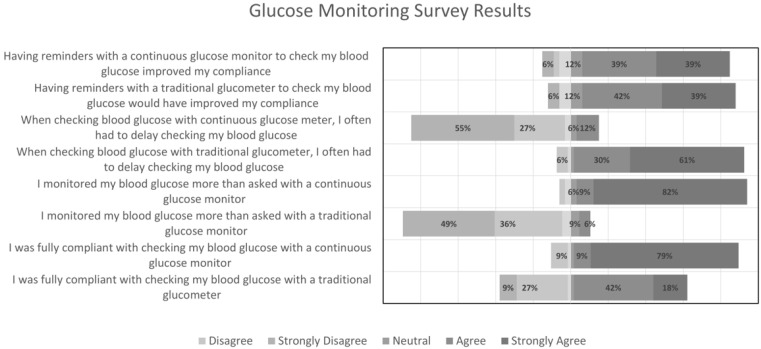
Glucose monitoring survey results.

A total of 33/35 students (95%) elected to submit the voluntary survey. Figure 1 displays the results of the domain regarding glucose monitoring and the comparison of the student experience between self-monitoring and the use of a CGM. Most students agreed that they had greater compliance when checking their blood glucose through utilization of the CGM compared to a glucometer (88% vs. 60%, respectively). There were also found to be fewer delays in checking their blood glucose with a CGM, as only 12% reported agreeing to delay a glucose check with a CGM compared to 91% with a glucometer. This could possibly be attributed to a CGM’s convenience of use and alerts, which many students agreed contributed to monitoring their blood glucose (78%). This may also be the reason students reported agreeing to check their blood glucose via CGM more than what was requested by the simulation as compared to a traditional glucometer (91% vs. 6%, respectively). Although many students agreed that they were compliant with checking their blood glucose via either means, more students strongly agreed when using the CGM.

**Figure 2:**
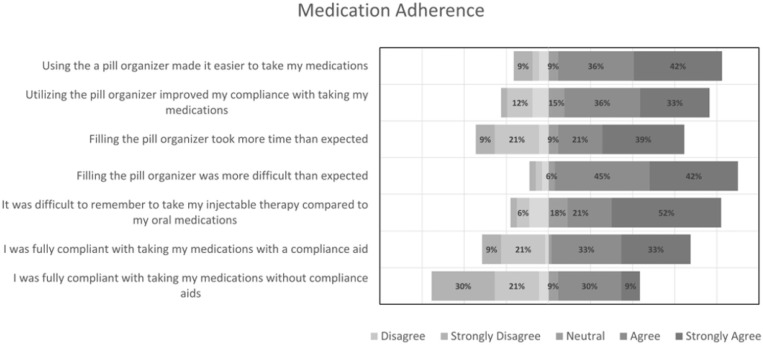
Medication adherence survey results.

Figure 2 displays the results from the medication adherence domain. Students noted that utilization of a pill organizer made it easier to take their medications (78%) while improving their compliance (69%). It also highlights other variables that students may not consider in an underserved patient population, such as the time and acuity required to set up the pill organizer, as most students realized it took more time (60%) and difficulty (87%) than they were anticipating. It also highlights the limitations of compliance aids, in this case, the pill organizer, as medications outside the constraints of this aid (i.e., injectable therapy) were reported as a difficulty among students (73%). Lastly, students reported that they were more compliant with their medications using a compliance aid than without (66% vs. 39%). For the last domain evaluated via survey to assess the overall implications of this experience, most participants (>80%) agreed that this project increased their understanding of the value of compliance aids and devices and encouraged them to not only incorporate them into their future patient care plans but also advocate for accessibility to improve health outcomes. Student reflections were evaluated via faculty and teaching assistants with many students receiving full credit for detailing their development of empathy and understanding of the simulation concepts and their applicability towards healthcare.

## 4. Discussion

This immersive simulation directly compared the patient’s experience of managing chronic disease states with and without compliance aids and devices. Previous studies, as mentioned previously, have shown improvements in students’ understanding of the devices themselves; however, these studies provided very little on the differences between using the device and not using the device [4,5,6,7,8,9,10,11,12,13,14,15,16]. This trial seems to reflect previous trials completed in that the students may become more aware of the proper use of such devices while adding more depth to the impact of not using such devices. Unfortunately, there is still a large portion of Americans without access to these compliance aids. However, it has been recently announced that Medicare will once again expand its coverage of CGMs to any person on insulin therapy and/or history of hypoglycemia [17]. With this change, along with reductions in insulin prices, it is crucial for student pharmacists to experience a real-world application of these devices [18]. With the evidence available showing little benefit of pill organizers, the results of this study may indicate that patient education, proper training, and improved health literacy may have a large impact on the usefulness of these devices. These results highlight a significant gap in healthcare that can profoundly impact some of the most vulnerable patients. One limitation was adequately assessing the adherence requirements to either medication administration or blood glucose monitoring for students without T2DM without having academic incentives as the sole driving force. As such, certain thresholds were made such as students were only required to acknowledge checking their blood glucose ≥ 60% of the time within the elective course site to receive full credit. For medication adherence, students had the same threshold and again were asked to acknowledge their adherence via the course site. However, there was no definitive way to see if blood glucose was physically checked and that medications were being administered by the students, as only acknowledgment via the course site was documented. Another potential limitation may be that the students first started the simulation as patients without the aid of any compliance devices. Many of the students have not been in a situation in which they themselves have to manage these disease states and complex medication regimens as patients. The second week of any simulation, regardless of the use of compliance aids, may prove to be less overwhelming as it is the second week of the simulation. However, this simulation only lasted over roughly 3 weeks which allows for very little time for this potential effect to take place. It is possible that even starting the immersive simulation with the compliance aids may have proven to be more difficult than anticipated.

The incorporation of the reflection assignment at the end of the course was designed in an attempt to combat this limitation, as students would have a difficult time reflecting on their experience if they did not immerse themselves within the simulation, but a limitation of the study, nonetheless.

## 5. Conclusions

The aim of this experience was to encourage future pharmacists to be empathetic towards patients struggling with chronic disease state management, understand the value and implementation of compliance aids and devices in the care of their patients, and spark the desire to become patient advocates. Students who completed this experience reported better adherence to chronic disease state control through the use of compliance aids and, in turn, the applicability of the use of compliance aids in managing those with complex medication regimens. This simulation may encourage these future pharmacists to incorporate compliance aids with their patients to improve health outcomes.

## Data Availability

Data are contained within the article.

## References

[1] Centers for Disease Control and Prevention Type 2 Diabetes. https://www.cdc.gov/diabetes/about/about-type-2-diabetes.html.

[2] American Diabetes Association (2022). Standards of Care in Diabetes-2023 Abridged for Primary Care Providers. Clin. Diabetes.

[3] American Diabetes Association Health Equity and Diabetes Technology: A Study of Access to Continuous Glucose Monitors by Payer and Race Executive Summary. Connect for Life 2021. https://diabetes.org/sites/default/files/2023-09/ADA-CGM-Utilization-White-Paper-Oct-2022.pdf.

[4] Korayem G.B., Alshaya O.A., Kurdi S.M., Alnajjar L.I., Badr A.F., Alfahed A., Cluntun A. (2022). Simulation-Based Education Implementation in Pharmacy Curriculum: A Review of the Current Status. Adv. Med. Educ. Pract..

[5] Dowden A. (2020). Do pill organisers improve medication adherence?. Prescriber.

[6] Darbishire P.L., Mashrah D. (2018). Comparison of Student and Patient Perceptions for Medication Non-adherence. Am. J. Pharm. Educ..

[7] Ulbrich T., Hamer D., Lehotsky K. (2012). Second-year pharmacy students’ perceptions of adhering to a complex simulated medication regimen. Am. J. Pharm. Educ..

[8] Witry M.J., LaFever M., Gu X. (2017). A Narrative Review of Medication Adherence Educational Interventions for Health Professions Students. Am. J. Pharm. Educ..

[9] Knezevich E., Fuji K.T., Larson K., Muniz G. (2022). A Cross-Sectional Survey Study Examining the Provision of Continuous Glucose Monitoring Education in U.S. Doctor of Pharmacy Programs. Pharmacy.

[10] Sherrill C.H., Lee S., Bradley C.L. (2022). Design and development of a continuous glucose monitoring educational module for students and practicing pharmacists. Curr. Pharm. Teach. Learn..

[11] Wirth F., Rivera R., Zaccomer A., Azzopardi L.M. (2022). Point-of-care testing devices and clinical skills practical sessions: A blended learning approach. Pharm. Educ..

[12] Morello C.M., Neighbors M., Luu L., Kobayashi S., Mutrux B., Best B.M. (2013). Impact of a first-year student pharmacist diabetes self-care education program. Am. J. Pharm. Educ..

[13] Delea D., Shrader S., Phillips C. (2010). A week-long diabetes simulation for pharmacy students. Am. J. Pharm. Educ..

[14] Parker D., Fontem A., Ojong E., Pope J. (2019). Impact of Diabetes Simulation on Empathy in Pharmacy Students. Am. J. Pharm. Educ..

[15] Whitley H.P. (2012). Active-learning diabetes simulation in an advanced pharmacy practice experience to develop patient empathy. Am. J. Pharm. Educ..

[16] Todd T.J., Mazan J.L., Stensland S.L. (2016). The impact of a type 2 diabetes, six-week immersion experience on adherence—A pilot study. Curr. Pharm. Teach. Learn..

[17] Chen S. (2023). Medicare Expands CGM Coverage for People with Type 2 Diabetes. https://diatribe.org/medicare-expands-cgm-continuous-glucose-monitor-coverage-type-2-diabetes.

[18] Berkeley L. (2023). For Many Insulin Users, New Price Cuts Will Be a ‘Lifeline’. https://www.nbcnews.com/health/health-news/insulin-users-respond-price-cuts-eli-lilly-novo-nordisk-sanofi-rcna75448.

